# Supplementary material for: COZOID: contact zone identifier for visual analysis of protein-protein interactions
Source: BMC Bioinformatics. 2018 Apr 6;19:125. doi: 10.1186/s12859-018-2113-6 (PMC5889581; doi:10.1186/s12859-018-2113-6)
Supplement: Supplementary file 2 — Software build. Executable binary file of the software tool. (ZIP 118,784 kb) [file 12859_2018_2113_MOESM2_ESM.zip › cozoid/java/docs/beansbinding-1.2.1-doc.zip!/overview-tree.html]

Class Hierarchy (Beans Binding)


---


|  |  |  |
| --- | --- | --- |
| **Overview** \| Package \| Class \| Use \| **Tree** \| **Deprecated** \| **Index** \| **Help** | |  |
| PREV   NEXT | **FRAMES**    **NO FRAMES**     **All Classes** |  |


---


## Hierarchy For All Packages

**Package Hierarchies:**: org.jdesktop.beansbinding, org.jdesktop.beansbinding.ext, org.jdesktop.el, org.jdesktop.el.impl, org.jdesktop.el.impl.lang, org.jdesktop.el.impl.parser, org.jdesktop.el.impl.util, org.jdesktop.observablecollections, org.jdesktop.swingbinding, org.jdesktop.swingbinding.adapters, org.jdesktop.swingbinding.impl

---

## Class Hierarchy

- java.lang.Object
  - org.jdesktop.beansbinding.**AbstractBindingListener** (implements org.jdesktop.beansbinding.BindingListener)- org.jdesktop.swingbinding.adapters.**AbstractButtonAdapterProvider** (implements org.jdesktop.beansbinding.ext.BeanAdapterProvider)- org.jdesktop.el.impl.parser.**AstValue.Target**- org.jdesktop.swingbinding.adapters.**BeanAdapterBase**
          - org.jdesktop.swingbinding.adapters.**AbstractButtonAdapterProvider.Adapter**- org.jdesktop.swingbinding.adapters.**JComboBoxAdapterProvider.Adapter**- org.jdesktop.swingbinding.adapters.**JListAdapterProvider.Adapter**- org.jdesktop.swingbinding.adapters.**JSliderAdapterProvider.Adapter**- org.jdesktop.swingbinding.adapters.**JSpinnerAdapterProvider.Adapter**- org.jdesktop.swingbinding.adapters.**JTableAdapterProvider.Adapter**- org.jdesktop.swingbinding.adapters.**JTextComponentAdapterProvider.Adapter**
          - org.jdesktop.beansbinding.ext.**BeanAdapterFactory**- org.jdesktop.el.**BeanELResolver.BeanProperties**- org.jdesktop.el.**BeanELResolver.BeanProperty**- org.jdesktop.beansbinding.**Binding**<SS,SV,TS,TV>
                  - org.jdesktop.swingbinding.impl.**AbstractColumnBinding**
                    - org.jdesktop.swingbinding.**JListBinding.DetailBinding**- org.jdesktop.swingbinding.**JTableBinding.ColumnBinding**
                    - org.jdesktop.beansbinding.**AutoBinding**<SS,SV,TS,TV>
                      - org.jdesktop.swingbinding.**JComboBoxBinding**<E,SS,TS>- org.jdesktop.swingbinding.**JListBinding**<E,SS,TS>- org.jdesktop.swingbinding.**JTableBinding**<E,SS,TS>
                  - org.jdesktop.beansbinding.**Binding.SyncFailure**- org.jdesktop.beansbinding.**Binding.ValueResult**<V>- org.jdesktop.beansbinding.**BindingGroup**- org.jdesktop.beansbinding.**Bindings**- org.jdesktop.beansbinding.**Converter**<S,T>- org.jdesktop.el.impl.lang.**ELArithmetic**
                              - org.jdesktop.el.impl.lang.**ELArithmetic.BigDecimalDelegate**- org.jdesktop.el.impl.lang.**ELArithmetic.BigIntegerDelegate**- org.jdesktop.el.impl.lang.**ELArithmetic.DoubleDelegate**- org.jdesktop.el.impl.lang.**ELArithmetic.LongDelegate**
                              - org.jdesktop.el.**ELContext**
                                - org.jdesktop.el.impl.lang.**EvaluationContext**
                                - org.jdesktop.el.impl.parser.**ELParser** (implements org.jdesktop.el.impl.parser.ELParserConstants, org.jdesktop.el.impl.parser.ELParserTreeConstants)- org.jdesktop.el.impl.parser.**ELParserTokenManager** (implements org.jdesktop.el.impl.parser.ELParserConstants)- org.jdesktop.el.**ELResolver**
                                      - org.jdesktop.el.**ArrayELResolver**- org.jdesktop.el.**BeanELResolver**- org.jdesktop.el.**CompositeELResolver**- org.jdesktop.el.**ListELResolver**- org.jdesktop.el.**MapELResolver**- org.jdesktop.el.**ResourceBundleELResolver**
                                      - org.jdesktop.el.impl.lang.**ELSupport**
                                        - org.jdesktop.el.impl.parser.**SimpleNode** (implements org.jdesktop.el.impl.parser.Node)
                                          - org.jdesktop.el.impl.parser.**ArithmeticNode**
                                            - org.jdesktop.el.impl.parser.**AstDiv**- org.jdesktop.el.impl.parser.**AstMinus**- org.jdesktop.el.impl.parser.**AstMod**- org.jdesktop.el.impl.parser.**AstMult**- org.jdesktop.el.impl.parser.**AstPlus**
                                            - org.jdesktop.el.impl.parser.**AstBracketSuffix**- org.jdesktop.el.impl.parser.**AstChoice**- org.jdesktop.el.impl.parser.**AstCompositeExpression**- org.jdesktop.el.impl.parser.**AstDeferredExpression**- org.jdesktop.el.impl.parser.**AstDotSuffix**- org.jdesktop.el.impl.parser.**AstDynamicExpression**- org.jdesktop.el.impl.parser.**AstEmpty**- org.jdesktop.el.impl.parser.**AstFloatingPoint**- org.jdesktop.el.impl.parser.**AstFunction**- org.jdesktop.el.impl.parser.**AstIdentifier**- org.jdesktop.el.impl.parser.**AstInteger**- org.jdesktop.el.impl.parser.**AstLiteralExpression**- org.jdesktop.el.impl.parser.**AstNegative**- org.jdesktop.el.impl.parser.**AstNot**- org.jdesktop.el.impl.parser.**AstNull**- org.jdesktop.el.impl.parser.**AstString**- org.jdesktop.el.impl.parser.**AstValue**- org.jdesktop.el.impl.parser.**BooleanNode**
                                                                                - org.jdesktop.el.impl.parser.**AstAnd**- org.jdesktop.el.impl.parser.**AstEqual**- org.jdesktop.el.impl.parser.**AstFalse**- org.jdesktop.el.impl.parser.**AstGreaterThan**- org.jdesktop.el.impl.parser.**AstGreaterThanEqual**- org.jdesktop.el.impl.parser.**AstLessThan**- org.jdesktop.el.impl.parser.**AstLessThanEqual**- org.jdesktop.el.impl.parser.**AstNotEqual**- org.jdesktop.el.impl.parser.**AstOr**- org.jdesktop.el.impl.parser.**AstTrue**
                                        - java.util.EventObject (implements java.io.Serializable)
                                          - org.jdesktop.el.**ELContextEvent**- org.jdesktop.beansbinding.**PropertyStateEvent**
                                          - org.jdesktop.el.**Expression** (implements java.io.Serializable)
                                            - org.jdesktop.el.**MethodExpression**
                                              - org.jdesktop.el.impl.**MethodExpressionImpl** (implements java.io.Externalizable)- org.jdesktop.el.impl.**MethodExpressionLiteral** (implements java.io.Externalizable)
                                              - org.jdesktop.el.**ValueExpression**
                                                - org.jdesktop.el.impl.**ValueExpressionImpl** (implements java.io.Externalizable)- org.jdesktop.el.impl.**ValueExpressionLiteral** (implements java.io.Externalizable)
                                            - org.jdesktop.el.**Expression.ResolvedProperty**- org.jdesktop.el.**Expression.Result**- org.jdesktop.el.impl.lang.**ExpressionBuilder** (implements org.jdesktop.el.impl.parser.NodeVisitor)- org.jdesktop.el.**ExpressionFactory**
                                                    - org.jdesktop.el.impl.**ExpressionFactoryImpl**
                                                    - org.jdesktop.el.**FunctionMapper**
                                                      - org.jdesktop.el.impl.lang.**FunctionMapperFactory**- org.jdesktop.el.impl.lang.**FunctionMapperImpl** (implements java.io.Externalizable)
                                                      - org.jdesktop.el.impl.lang.**FunctionMapperImpl.Function** (implements java.io.Externalizable)- org.jdesktop.swingbinding.adapters.**JComboBoxAdapterProvider** (implements org.jdesktop.beansbinding.ext.BeanAdapterProvider)- org.jdesktop.swingbinding.adapters.**JListAdapterProvider** (implements org.jdesktop.beansbinding.ext.BeanAdapterProvider)- org.jdesktop.swingbinding.adapters.**JSliderAdapterProvider** (implements org.jdesktop.beansbinding.ext.BeanAdapterProvider)- org.jdesktop.swingbinding.adapters.**JSpinnerAdapterProvider** (implements org.jdesktop.beansbinding.ext.BeanAdapterProvider)- org.jdesktop.swingbinding.adapters.**JTableAdapterProvider** (implements org.jdesktop.beansbinding.ext.BeanAdapterProvider)- org.jdesktop.swingbinding.adapters.**JTextComponentAdapterProvider** (implements org.jdesktop.beansbinding.ext.BeanAdapterProvider)- org.jdesktop.swingbinding.impl.**ListBindingManager** (implements org.jdesktop.observablecollections.ObservableListListener)- org.jdesktop.el.impl.util.**MessageFactory**- org.jdesktop.el.**MethodInfo**- org.jdesktop.observablecollections.**ObservableCollections**- org.jdesktop.observablecollections.**ObservableCollections.ObservableListHelper**<E>- org.jdesktop.beansbinding.**Property**<S,V>
                                                                                - org.jdesktop.beansbinding.**ObjectProperty**<S>- org.jdesktop.beansbinding.**PropertyHelper**<S,V>
                                                                                    - org.jdesktop.beansbinding.**BeanProperty**<S,V>- org.jdesktop.beansbinding.**ELProperty**<S,V>
                                                                                - org.jdesktop.el.impl.util.**ReflectionUtil**- org.jdesktop.el.impl.parser.**SimpleCharStream**- org.jdesktop.swingbinding.**SwingBindings**- java.lang.Throwable (implements java.io.Serializable)
                                                                                        - java.lang.Error
                                                                                          - org.jdesktop.el.impl.parser.**TokenMgrError**
                                                                                          - java.lang.Exception
                                                                                            - org.jdesktop.el.impl.parser.**ParseException**- java.lang.RuntimeException
                                                                                                - org.jdesktop.el.**ELException**
                                                                                                  - org.jdesktop.el.**MethodNotFoundException**- org.jdesktop.el.**PropertyNotFoundException**- org.jdesktop.el.**PropertyNotWritableException**
                                                                                                  - org.jdesktop.beansbinding.**PropertyResolutionException**
                                                                                        - org.jdesktop.el.impl.parser.**Token**- org.jdesktop.beansbinding.**Validator**<T>- org.jdesktop.beansbinding.**Validator.Result**- org.jdesktop.el.**VariableMapper**
                                                                                                - org.jdesktop.el.impl.lang.**VariableMapperFactory**- org.jdesktop.el.impl.lang.**VariableMapperImpl** (implements java.io.Externalizable)

## Interface Hierarchy

- org.jdesktop.beansbinding.ext.**BeanAdapterProvider**- org.jdesktop.el.impl.parser.**ELParserConstants**- org.jdesktop.el.impl.parser.**ELParserTreeConstants**- java.util.EventListener
        - org.jdesktop.beansbinding.**BindingListener**- org.jdesktop.el.**ELContextListener**- org.jdesktop.observablecollections.**ObservableListListener**- org.jdesktop.beansbinding.**PropertyStateListener**
        - java.lang.Iterable<T>
          - java.util.Collection<E>
            - java.util.List<E>
              - org.jdesktop.observablecollections.**ObservableList**<E>
              - org.jdesktop.observablecollections.**ObservableList**<E>
            - java.util.List<E>
              - org.jdesktop.observablecollections.**ObservableList**<E>
              - org.jdesktop.observablecollections.**ObservableList**<E>
          - java.util.Map<K,V>
            - org.jdesktop.observablecollections.**ObservableMap**<K,V>
            - org.jdesktop.el.impl.parser.**Node**- org.jdesktop.el.impl.parser.**NodeVisitor**- org.jdesktop.observablecollections.**ObservableMapListener**

## Enum Hierarchy

- java.lang.Object
  - java.lang.Enum<E> (implements java.lang.Comparable<T>, java.io.Serializable)
    - org.jdesktop.beansbinding.**Binding.SyncFailureType**- org.jdesktop.beansbinding.**AutoBinding.UpdateStrategy**- org.jdesktop.el.**Expression.Result.Type**

---


|  |  |  |
| --- | --- | --- |
| **Overview** \| Package \| Class \| Use \| **Tree** \| **Deprecated** \| **Index** \| **Help** | |  |
| PREV   NEXT | **FRAMES**    **NO FRAMES**     **All Classes** |  |


---
